# Two new species of the genus *Sinocrassula* (Crassulaceae) from Yunnan Province, China

**DOI:** 10.3897/phytokeys.278.190330

**Published:** 2026-07-27

**Authors:** Sheng-Rong Gong, Jing Zhao, Jian-Rong Zhang, Shi-Feng Liang, Ling-Nan Wei, Xin-Mao Zhou, Jia-Guan Wang

**Affiliations:** 1 School of Life Sciences & School of Ecology and Environmental Science, Yunnan University, Kunming, 650504, Yunnan, China School of Life Sciences & School of Ecology and Environmental Science, Yunnan University Kunming China

**Keywords:** Morphology, nuclear genes, phylogeny, taxonomy, Telephium clade

## Abstract

Based on comprehensive morphological and molecular data analyses, this study confirms that three populations of *Sinocrassula* from central Yunnan Province represent two distinct new species, which are described herein as *Sinocrassula kunmingensis* J.Guan Wang & Jing Zhao and *S. chenggongensis* J.Guan Wang & Jing Zhao. Morphologically, the two new species are most similar to *S. adpressa*, *S. crassifolia*, *S. obliquifolia*, and *S. yongshengensis*. However, *S. kunmingensis* is characterized by its greenish-yellow or red stem leaves without spots, subligulate nectar scales, reddish-purple flowers, ovate-lanceolate bracts, and petals with a slight central protrusion; *S. chenggongensis* is distinguished by its green stem leaves with purplish-red spots, subligulate nectar scales, pinkish-purple flowers, oblong-elliptic bracts with an oblique apex, and petals without a protrusion. This combination of morphological characters can be clearly distinguished from those of similar species. Phylogenetic analyses further reveal that the two new species form two independent clades with strong support and are sister to each other.

## Introduction

*Sinocrassula* A. Berger belongs to the family Crassulaceae J. St.-Hil. of Saxifragales Bercht. & J. Presl (Thiede and Eggli 2007; Messerschmid et al. 2020). The genus was established by Berger (1930) based on *Sinocrassula indica* (Decne.) A. Berger and is primarily distributed in the Himalaya-Hengduan Mountains (Zhao et al. 2025). In addition, *Sinocrassula* represents one of the most morphologically and ecologically diverse lineages within the Telephium clade of Sempervivoideae (Zhao et al. 2025). The main diagnostic characteristics of *Sinocrassula* include pentamerous flowers, a single whorl of stamens, carpels connate at the base, and basal leaves usually arranged in a rosette (Berger 1930). Some species of the genus possess both horticultural and medicinal values (He et al. 2025; Zhao et al. 2025). For example, *S. yunnanensis* A. Berger is widely cultivated as a succulent plant and popular in horticultural markets (Zhao et al. 2025), whereas the methanol extract of *S. indica* significantly reduces blood glucose levels in animals, delays gastric emptying, has a potent hepatoprotective effect (Yoshikawa et al. 2007; Ninomiya et al. 2008), and is often used to treat rheumatic arthritis, stomachache, and fractures (Zhao et al. 2004; Xie and Yoshikawa 2012). In recent years, ten new species of *Sinocrassula* have been reported across Asia (Wang et al. 2012; Averyanov et al. 2014; Wang et al. 2022; Li et al. 2024, 2025; Qiu et al. 2025; Xu et al. 2025; He et al. 2025; Zhao et al. 2026). The continuous discovery of new species not only provides essential material for systematic and evolutionary studies but also supplies potential new medicinal resources (Zhao et al. 2004; Xie and Yoshikawa 2012).

In June 2023 and October 2025, two field surveys were conducted in the areas surrounding Qiongzhu Temple in Wuhua District and Santai Mountain in Chenggong District, Kunming City, Yunnan Province, respectively, during which three natural populations of *Sinocrassula* were discovered. Through a literature review, cultivation observation, and morphological comparison, these three populations were confirmed to belong to two species and to be morphologically different from each other and significantly distinct from all known species within *Sinocrassula*. Here, the taxonomic status of these three populations is examined based on morphological observations and molecular phylogenetic analyses, and they are described as two new species of *Sinocrassula*.

## Materials and methods

### Morphological study

Specimens of the new species were collected from Kunming City, Yunnan Province, China, and vouchers were deposited in the Herbarium of Yunnan University (YUKU). Living plants were cultivated in the greenhouse at Yunnan University, and their morphological features, including leaves, inflorescences, and flowers, were observed and documented using an SMZ1270 stereo microscope (Nikon, Japan). Mature pollen grains were selected using an anatomical needle under a stereomicroscope, mounted on carbon adhesive tape-coated specimen stubs, and their color was recorded. The stubs were sputter-coated with gold for 5 min using a BAL-TEC SCD 005 ion coater. Pollen morphology and surface ornamentation were examined under a QUANTA 200 scanning electron microscope (SEM). Morphological terminology for pollen follows Paul et al. (2022). To confirm the restricted distribution of the new species and ensure that no relevant specimens were overlooked, the following online databases were searched: Chinese Virtual Herbarium (CVH, http://www.cvh.ac.cn), National Specimen Information Infrastructure (NSII, http://www.nsii.org.cn), JSTOR Global Plants (https://plants.jstor.org), and the online collections of the Herbarium of Kunming Institute of Botany (KUN). No additional specimens of the two new species were found beyond those reported in this study.

### Taxonomic sampling

To explore the phylogenetic positions of the new species, the phylogenetic analyses included three samples of the new species from different populations, along with their putative relatives from the genus *Sinocrassula* in China and surrounding regions. In total, 54 specimens representing 23 species of *Sinocrassula* were included in the phylogenetic analyses (Table 1). Based on previous phylogenetic studies (e.g., Zhao et al. 2025), species of *Kungia* K.T. Fu were selected as the outgroups.

**Table 1.** List of taxa sampled in this study.

Species	Voucher	*trn*L-*trn*F	*psb*A-*trn*H	*rbc*L	*mat*K	ITS	Reference
*Kungia aliciae* (Raym.-Hamet) K.T. Fu	CH00061 (TI)	AB480632	–	–	–	AB480591	Mayuzumi and Ohba 2009
*Kungia aliciae* var. *komarovii* (Raym.-Hamet) K.T. Fu	Chen s.n. (YUKU)	PV352314	PV352314	PV352314	PV352314	PX778028	Zhao et al. 2025, 2026
*Sinocrassula adpressa* J.Guan Wang	YUS9494 (YUKU)	PV052278	PV052280	PV052285	PV052290	PV033531	He et al. 2025
*Sinocrassula adpressa* J.Guan Wang	YUS9570 (YUKU)	PV052277	PV052279	PV052284	PV052289	PV033530	He et al. 2025
*Sinocrassula ambigua* (Praeger) A. Berger	YUS12672 (YUKU)	PQ629030	PQ629055	PQ629038	PQ629046	PQ611188	Xu et al. 2025
*Sinocrassula ambigua* (Praeger) A. Berger	YUS12973 (YUKU)	PQ629032	PQ629054	PQ629039	PQ629047	PQ611189	Xu et al. 2025
*Sinocrassula ambigua* (Praeger) A. Berger	YUS6698 (YUKU)	PQ629035	PQ629059	PQ629040	PQ629048	PQ611190	Xu et al. 2025
*Sinocrassula chenggongensis* J.Guan Wang & Jing Zhao	YUS15602 (YUKU)	PZ380325	PZ380322	PZ380319	PZ380316	PZ381590	this study
*Sinocrassula crassifolia* Chao Chen, Jing Zhao & J.Guan Wang	YUS11746 (YUKU)	PV352322	PV352322	PV352322	PV352322	PX778029	Zhao et al. 2025, 2026
*Sinocrassula crassifolia* Chao Chen, Jing Zhao & J.Guan Wang	YUS11994 (YUKU)	PV352321	PV352321	PV352321	PV352321	PX778030	Zhao et al. 2025, 2026
*Sinocrassula crassifolia* Chao Chen, Jing Zhao & J.Guan Wang	YUS14579 (YUKU)	PV352320	PV352320	PV352320	PV352320	PX778052	Zhao et al. 2025, 2026
*Sinocrassula cuonaensis* N. Wei & Q.F. Wang	Wei & Xin WN43 (HIB, PE)	PV641595	PV641593	PV610839	PV610727	PV548041	Qiu et al. 2025
*Sinocrassula densirosulata* (Praeger) A. Berger	SZXC19075	MW206800	MW206800	MW206800	MW206800	–	Unknown
*Sinocrassula densirosulata* (Praeger) A. Berger	YUS11971 (YUKU)	PV052276	PV052283	PV052286	PV052291	PV033527	He et al. 2025
*Sinocrassula diversifolia* H. Chuang	YUS9407 (YUKU)	PQ629070	PQ629074	PQ629066	PQ629062	PQ623396	Li et al. 2025
*Sinocrassula diversifolia* H. Chuang	YUS9477 (YUKU)	PQ629071	PQ629075	PQ629067	PQ629063	PQ623397	Li et al. 2025
*Sinocrassula ganluoensis* R.J. Li, Jing Zhao & J.Guan Wang	YUS13920 (YUKU)	PQ505692	PQ505694	PQ505696	PQ505698	PQ496499	Li et al. 2024
*Sinocrassula ganluoensis* R.J. Li, Jing Zhao & J.Guan Wang	YUS6699 (YUKU)	PQ505691	PQ505693	PQ505695	PQ505697	PQ496498	Li et al. 2024
*Sinocrassula holotricha* J.Guan Wang, Jing Zhao & Chao Chen	YUS12867 (YUKU)	PQ629031	PQ629057	PQ629043	PQ629051	PQ611193	Xu et al. 2025
*Sinocrassula holotricha* J.Guan Wang, Jing Zhao & Chao Chen	YUS13475 (YUKU)	PQ629034	PQ629056	PQ629042	PQ629050	PQ611192	Xu et al. 2025
*Sinocrassula indica* (Decne.) A. Berger	YUS14550 (YUKU)	PV352330	PV352330	PV352330	PV352330	PX778041	Zhao et al. 2025, 2026
*Sinocrassula indica* (Decne.) A. Berger	YUS9066 (YUKU)	PV052275	PV052282	PV052288	PV052293	PV033529	He et al. 2025
*Sinocrassula indica* (Decne.) A. Berger	YUS9367 (YUKU)	PV052274	PV052281	PV052287	PV052292	PV033528	He et al. 2025
*Sinocrassula indica* var. *forrestii* (Raym.-Hamet) H. Chuang	YUS14440 (YUKU)	PV352332	PV352332	PV352332	PV352332	PX778034	Zhao et al. 2025, 2026
*Sinocrassula indica* var. *forrestii* (Raym.-Hamet) H. Chuang	YUS14578 (YUKU)	PV352333	PV352333	PV352333	PV352333	PX778051	Zhao et al. 2025, 2026
*Sinocrassula indica* var. *luteorubra* (Praeger) S.H. Fu	YUS14431 (YUKU)	PV352334	PV352334	PV352334	PV352334	PX778032	Zhao et al. 2025, 2026
*Sinocrassula indica* var. *luteorubra* (Praeger) S.H. Fu	YUS14432 (YUKU)	PV352335	PV352335	PV352335	PV352335	PX778033	Zhao et al. 2025, 2026
*Sinocrassula indica* var. *luteorubra* (Praeger) S.H. Fu	YUS14577 (YUKU)	PV352336	PV352336	PV352336	PV352336	PX778050	Zhao et al. 2025, 2026
*Sinocrassula indica* var. *obtusifolia* (Fröd.) S.H. Fu	YUS13936 (YUKU)	PQ505699	PQ505701	PQ505703	PQ505705	PQ496500	Li et al. 2024
*Sinocrassula indica* var. *obtusifolia* (Fröd.) S.H. Fu	YUS13959 (YUKU)	PQ505700	PQ505702	PQ505704	PQ505706	PQ496501	Li et al. 2024
*Sinocrassula indica* var. *serrata* (Raym.-Hamet) S.H. Fu	YUS14575 (YUKU)	PV352339	PV352339	PV352339	PV352339	PX778048	Zhao et al. 2025, 2026
*Sinocrassula indica* var. *serrata* (Raym.-Hamet) S.H. Fu	YUS14576 (YUKU)	PV352340	PV352340	PV352340	PV352340	PX778049	Zhao et al. 2025, 2026
*Sinocrassula jiaozishanensis* Chao Chen, J.Guan Wang & Z.R. He	JZS001 (YUKU)	MZ343264	MZ343262	MZ343263	MZ343261	–	Wang et al. 2022
*Sinocrassula jiaozishanensis* Chao Chen, J.Guan Wang & Z.R. He	JZS002 (YUKU)	MZ343269	MZ343267	MZ343268	MZ343266	–	Wang et al. 2022
*Sinocrassula jiaozishanensis* Chao Chen, J.Guan Wang & Z.R. He	YUS5900 (YUKU)	PQ629036	PQ629058	PQ629044	PQ629052	PQ611194	Xu et al. 2025
*Sinocrassula kunmingensis* J.Guan Wang & Jing Zhao	YUS15397 (YUKU)	PZ380327	PZ380323	PZ380320	PZ380318	PZ381591	this study
*Sinocrassula kunmingensis* J.Guan Wang & Jing Zhao	YUS15601 (YUKU)	PZ380326	PZ380324	PZ380321	PZ380317	–	this study
*Sinocrassula longistyla* (Praeger) S.H. Fu	YUS14558 (YUKU)	PV352343	PV352343	PV352343	PV352343	PX778043	Zhao et al. 2025, 2026
*Sinocrassula obliquifolia* Jing Zhao, J.Guan Wang & X.M. Zhou	YUS9064 (YUKU)	PQ629072	PQ629076	PQ629068	PQ629064	PQ623398	Li et al. 2025
*Sinocrassula obliquifolia* Jing Zhao, J.Guan Wang & X.M. Zhou	YUS9366 (YUKU)	PQ629073	PQ629077	PQ629069	PQ629065	PQ623399	Li et al. 2025
*Sinocrassula stenosquamata* Jian Wang & F. Du	YUS14481 (YUKU)	PV352347	PV352347	PV352347	PV352347	PX778039	Zhao et al. 2025, 2026
*Sinocrassula stenosquamata* Jian Wang & F. Du	YUS14567 (YUKU)	PV352348	PV352348	PV352348	PV352348	PX778045	Zhao et al. 2025, 2026
*Sinocrassula techinensis* (S.H. Fu) S.H. Fu	YUS14427 (YUKU)	PV352349	PV352349	PV352349	PV352349	PX778031	Zhao et al. 2025, 2026
*Sinocrassula techinensis* (S.H. Fu) S.H. Fu	YUS14462 (YUKU)	PV352350	PV352350	PV352350	PV352350	PX778036	Zhao et al. 2025, 2026
*Sinocrassula techinensis* (S.H. Fu) S.H. Fu	YUS14557 (YUKU)	PV352351	PV352351	PV352351	PV352351	PX778042	Zhao et al. 2025, 2026
*Sinocrassula techinensis* (S.H. Fu) S.H. Fu	YUS14566 (YUKU)	PV352352	PV352352	PV352352	PV352352	PX778044	Zhao et al. 2025, 2026
*Sinocrassula techinensis* (S.H. Fu) S.H. Fu	YUS14574 (YUKU)	PV352353	PV352353	PV352353	PV352353	PX778047	Zhao et al. 2025, 2026
*Sinocrassula vietnamensis* Aver. & V.V. Byalt	YUS14568 (YUKU)	PV352355	PV352355	PV352355	PV352355	PX778046	Zhao et al. 2025, 2026
*Sinocrassula vietnamensis* Aver. & V.V. Byalt	YUS14479 (YUKU)	PV352354	PV352354	PV352354	PV352354	PX778038	Zhao et al. 2025, 2026
*Sinocrassula yongshengensis* Chao Chen & J.Guan Wang	YUS16341 (YUKU)	PV983442	PV983442	PV983442	PV983442	PX973555	Zhao et al. 2026
*Sinocrassula yunnanensis* (Franch.) A. Berger	L.Y. Chen; HIB (cult.)	–	–	–	KC988295	KC988288	Chen et al. 2014
*Sinocrassula yunnanensis* (Franch.) A. Berger	Mayuzumi C00115 (TI)	AB480669	–	–	–	AB088582	Mayuzumi and Ohba 2004
*Sinocrassula yunnanensis* (Franch.) A. Berger	YUS13776 (YUKU)	PQ629033	PQ629061	PQ629041	PQ629049	PQ611191	Xu et al. 2025
*Sinocrassula yunnanensis* (Franch.) A. Berger	YUS14441 (YUKU)	PV352356	PV352356	PV352356	PV352356	PX778035	Zhao et al. 2025, 2026
*Sinocrassula yunnanensis* (Franch.) A. Berger	YUS14484 (YUKU)	PV352357	PV352357	PV352357	PV352357	PX778040	Zhao et al. 2025, 2026
*Sinocrassula yunnanensis* (Franch.) A. Berger	YUS6697 (YUKU)	PQ629037	PQ629060	PQ629045	PQ629053	PQ611195	Xu et al. 2025

### DNA extraction, amplification, and sequencing

Total genomic DNA was extracted from silica-dried materials using a TIANGEN plant genomic DNA extraction kit (TIANGEN Biotech, Beijing, China), strictly following the manufacturer’s instructions. In this study, four chloroplast markers [Sang et al. (1997, *psbA-trnH*), Taberlet et al. (1991, *trnL-F*), Kress and Erickson (2007, *rbcL*), Hollingsworth et al. (2009, *matK*)] and one nuclear marker [Mayuzumi et al. (2004, ITS)] were amplified and sequenced. PCR products were purified and sequenced by TSINGKE Biological Technology (Chengdu, China). Sequence data were examined using Sequencher v4.1.2 (Gene Codes Corporation, Ann Arbor, MI, USA) to check chromatograms for sequence validity, followed by editing and assembly in Geneious v11.1.4 (Kearse et al. 2012).

### Phylogenetic analyses

Sequences newly generated in this study and those downloaded from GenBank were aligned using MAFFT v7.450 (Katoh and Standley 2013). The alignment for each marker was imported into BioEdit v7.7.1.0 (Hall 1999) for manual adjustment and refinement, then exported in FASTA format. A combined matrix was constructed by concatenating the nuclear (ITS) and chloroplast fragments (*psbA-trnH*, *trnL-F*, *rbcL*, *matK*) using PhyloSuite v1.2.2 (Zhang et al. 2020). Maximum likelihood (ML, Felsenstein 1973) tree search and ML bootstrapping (BS) analysis were conducted on the CIPRES Science Gateway using RAxML-HPC2 (Miller et al. 2010). The analysis employed the default GTRCAT model based on the concatenated dataset, with 5,000 rapid bootstrap replicates followed by a search for the best-scoring tree in a single run (Stamatakis et al. 2008). Bayesian inference (BI) analysis was performed using MrBayes v3.2.7 (Huelsenbeck and Ronquist 2001) with the GTR+F+I+G4 model. Two independent runs were conducted, each with four Markov chain Monte Carlo chains (one cold, three heated), starting from a random tree and sampling one tree every 1,000 generations over 20,000,000 generations. Convergence and stationarity were assessed using Tracer v1.4 (Rambaut and Drummond 2007), and the first 25% of trees were discarded as burn-in. The remaining trees were used to calculate a 50% majority-rule consensus tree and posterior probabilities (PP). The resulting phylogenetic trees were visualized and formatted in FigTree v1.4.3 (Rambaut 2017).

## Results

**Figure 1.**
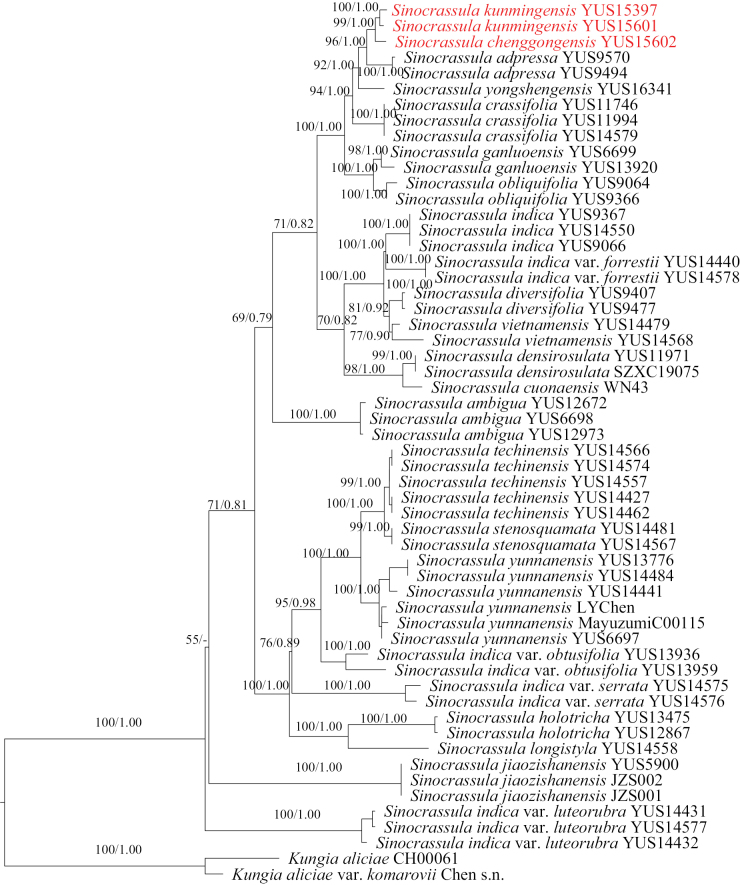
Maximum likelihood phylogeny of *Sinocrassula* and its allies based on four chloroplast markers (*psbA-trnH*, *trnL-F*, *rbcL*, and *matK*) and one nuclear marker (ITS). Values associated with branches are maximum likelihood bootstrap support (ML-BS) and Bayesian inference posterior probability (BI-PP).

A total of 267 sequences were used in this study, including 53 *rbcL*, 53 *psbA-trnH*, 55 *trnL-F*, 54 *matK*, and 52 ITS sequences, of which 14 sequences were newly generated for this study. Voucher information and GenBank accession numbers for all sequences are provided in Table 1. The tree topologies obtained from ML and BI based on the concatenated dataset were generally concordant. The phylogenetic tree is shown in Fig. 1.

In the phylogeny, each of the new species formed a highly supported monophyletic clade (ML-BS = 100; BI-PP = 1.00, Fig. 1), and they were resolved as sister to each other with strong support (ML-BS = 99; BI-PP = 1.00, Fig. 1). These two new species were then recovered together as sisters to *Sinocrassula adpressa* J.Guan Wang (ML-BS = 96; BI-PP = 1.00, Fig. 1). Morphologically, the main differences between the new species and their relatives are as follows: *S. kunmingensis* has stem leaves without spots and petals with a slight central protrusion, whereas *S. chenggongensis* has green stem leaves with purplish-red spots, oblong-elliptic bracts, and petals without a protrusion. A detailed morphological comparison is provided in Table 2. Based on the combined evidence from morphology and molecular phylogeny, they are proposed as two new species of *Sinocrassula*.

**Table 2.** Morphological comparison of *Sinocrassula kunmingensis*, *S. chenggongensis*, *S. crassifolia*, *S. obliquifolia*, *S. yongshengensis*, and *S. adpressa*.

Characters	*Sinocrassula kunmingensis*	*S. chenggongensis*	*S. crassifolia*	*S. obliquifolia*	*S. yongshengensis*	*S. adpressa*
Life cycle	Perennial	Perennial	Perennial	Perennial	Perennial	Perennial
Basal leaves	Rosulate, asymmetrically long-spatulate	Rosulate, asymmetrically obovate-lanceolate	Rosulate, pear-shaped or spindle-shaped	Rosulate, asymmetrically ovoid to lanceolate	Rosulate, spatulate-oblong	Rosulate, spatulate
Plant surface	Glabrous	Glabrous	Glabrous	Glabrous	Glabrous	Glabrous
Stem leaves	Greenish yellow or red without spots	Green with purplish-red spots	Purple red	Purple red, many parts with purple spots	—	Purple red with spots
Bracts	Ovate-lanceolate	Oblong-elliptic	Lanceolate	Lanceolate	Spatulate or ovate-oblong	Elliptic
Inflorescences	10–15 cm	12–15 cm	3–10 cm	10–18 cm	4.5–15 cm	8–20 cm
Flowers color	Reddish purple	Pinkish purple	Pinkish white	Reddish purple	White to green	Reddish purple
Petals	Central with a slight protrusion	Without a protrusion	Without a protrusion	Without a protrusion	Upper middle with a ridged protrusion	Central with a protrusion
Nectar scales	Subligulate	Subligulate	Reniform	Rectangular	Rectangular	Rectangular
Nectar scale sizes	0.5–0.8 × 0.4–0.8 mm	0.5–0.8 × 0.4–0.6 mm	0.5–1.0 × 0.4–0.5 mm	0.3–0.5 × 0.2–0.3 mm	0.4–0.7 × 0.3–0.5 mm	0.5–0.7 × 0.3–0.5 mm
Phenology	Sep.–Jan.	Sep.–Jan.	Apr.–Jun.	Jun.–Oct.	Aug.–Oct.	Sep.–Nov.

## Discussion

This study reports two new species of *Sinocrassula* distributed in Yunnan Province, China. Molecular phylogenetic analyses indicated that the two species are sister taxa and are closely related to *S. adpressa* (Fig. 1). The discovery of these two new species further reveals the species diversity of *Sinocrassula* in China and provides new biological material for exploring their potential medicinal and ornamental value. Furthermore, metabolomic and functional validation studies could be conducted on the potential bioactive compounds of these two new species, thereby promoting their sustainable development and applications in the medicinal and horticultural fields.

**Figure 2.**
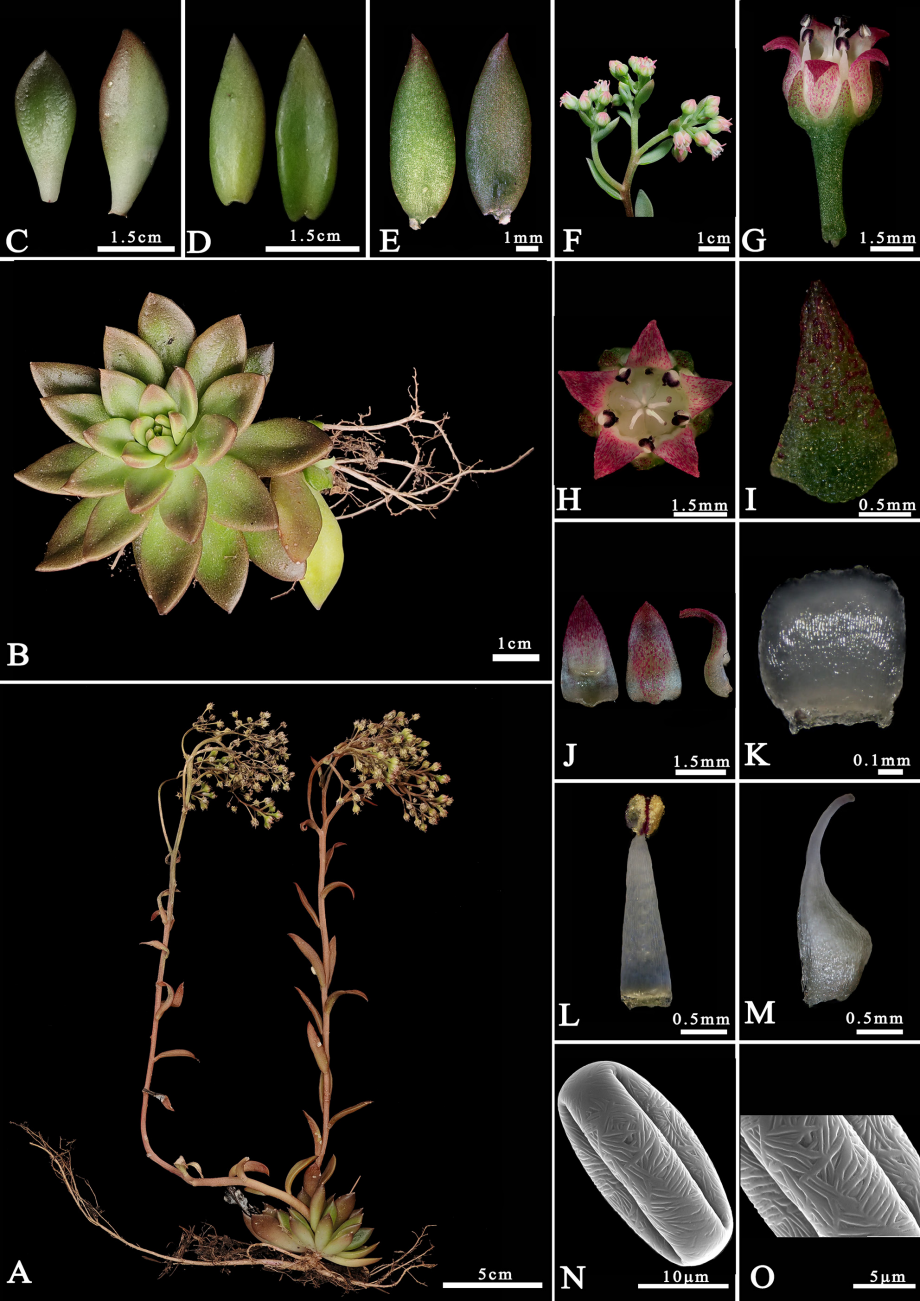
*Sinocrassula kunmingensis*. **A, B**. Habit; **C**. Basal leaves; **D**. Stem leaves; **E**. Bracts; **F–H**. Flowers; **I**. Sepal; **J**. Petals; **K**. Nectar scale; **L**. Stamen; **M**. Carpel; **N, O**. Pollen.

**Figure 3.**
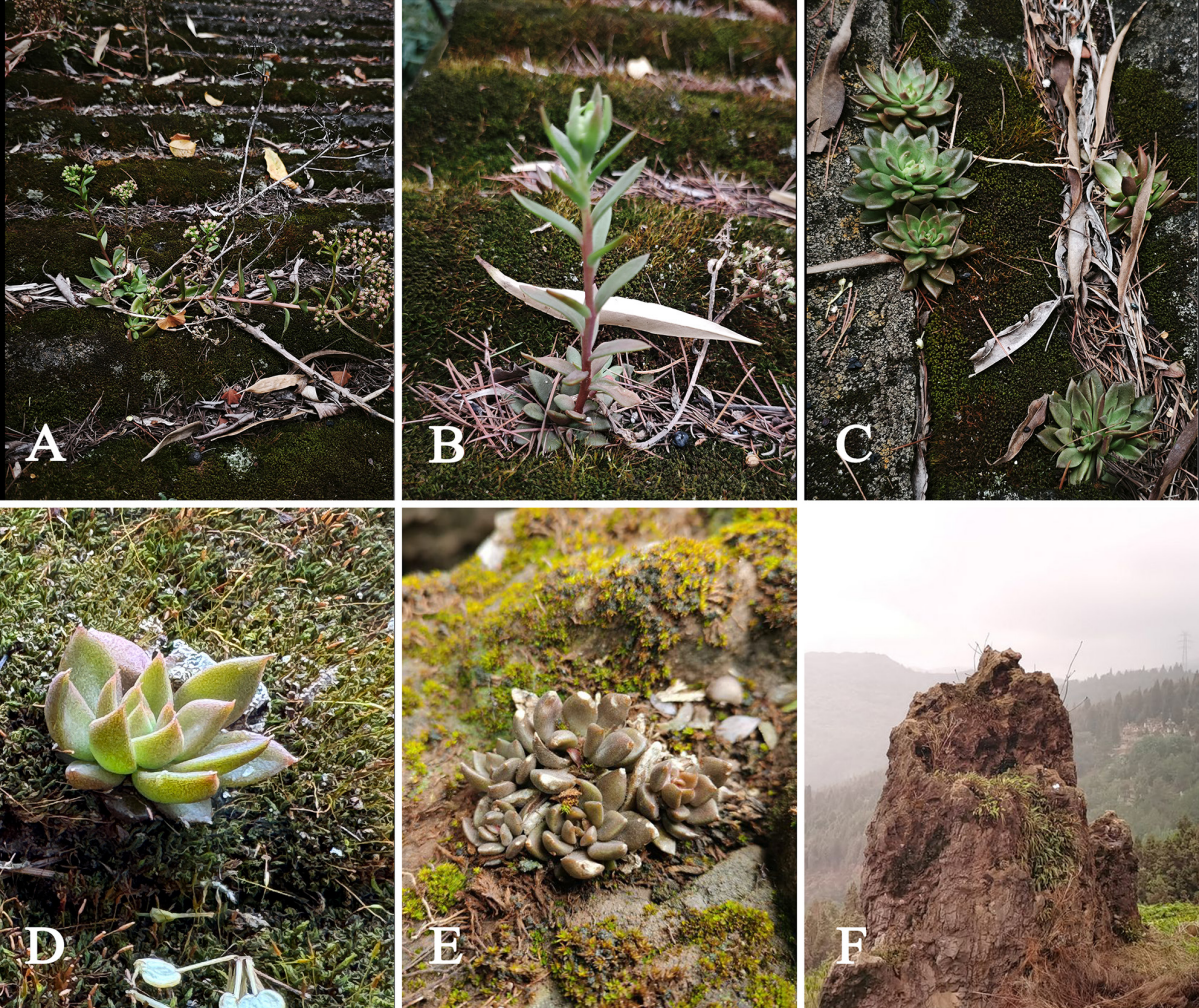
*Sinocrassula kunmingensis*. **A–D**. Shaded habit; **E**. Sun-exposed habit; **F**. Habitat.

**Figure 4.**
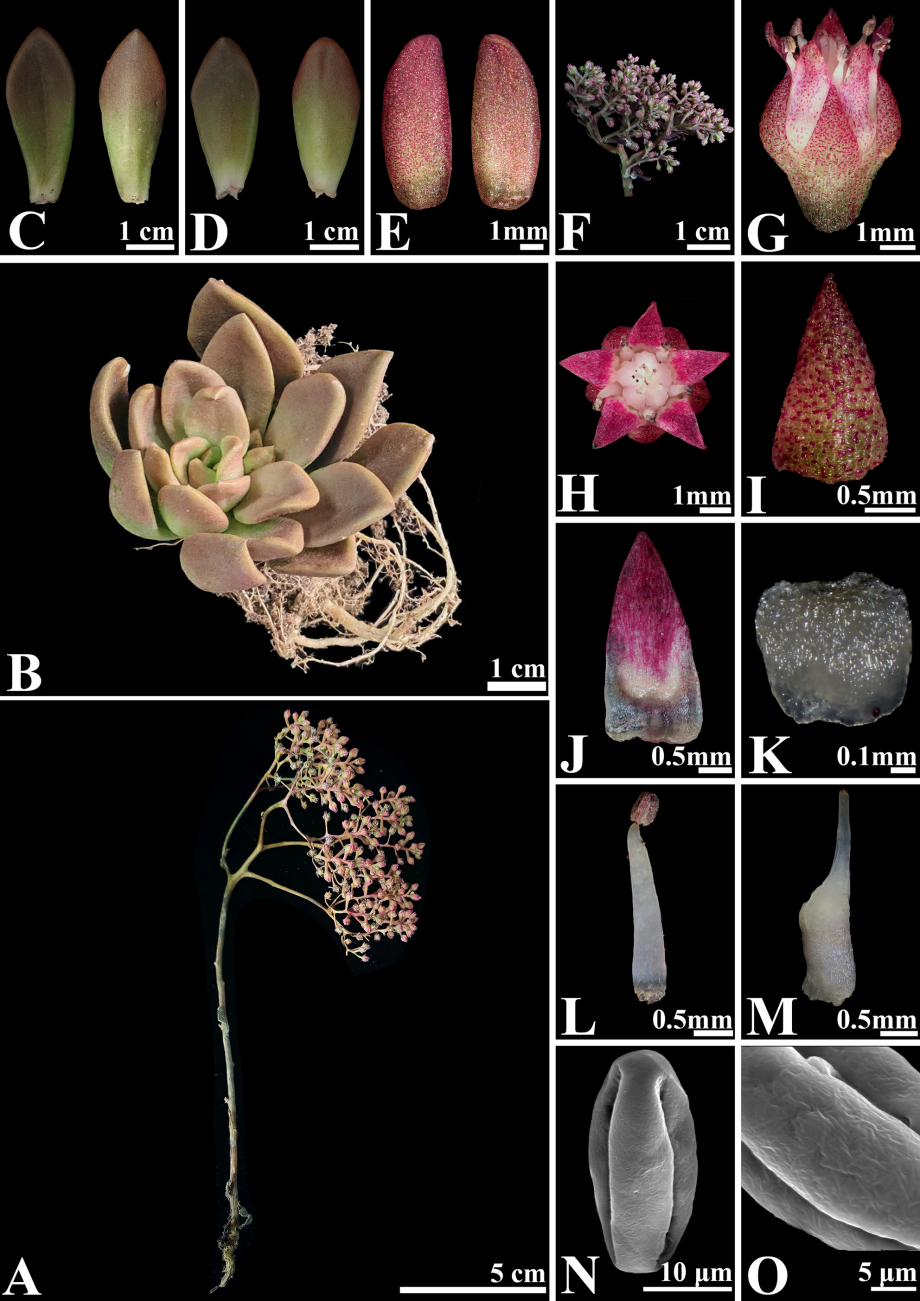
*Sinocrassula chenggongensis*. **A, B**. Habit; **C**. Basal leaves; **D**. Stem leaves; **E**. Bracts; **F–H**. Flowers; **I**. Sepal; **J**. Petal; **K**. Nectar scale; **L**. Stamen; **M**. Carpel; **N, O**. Pollen.

**Figure 5.**
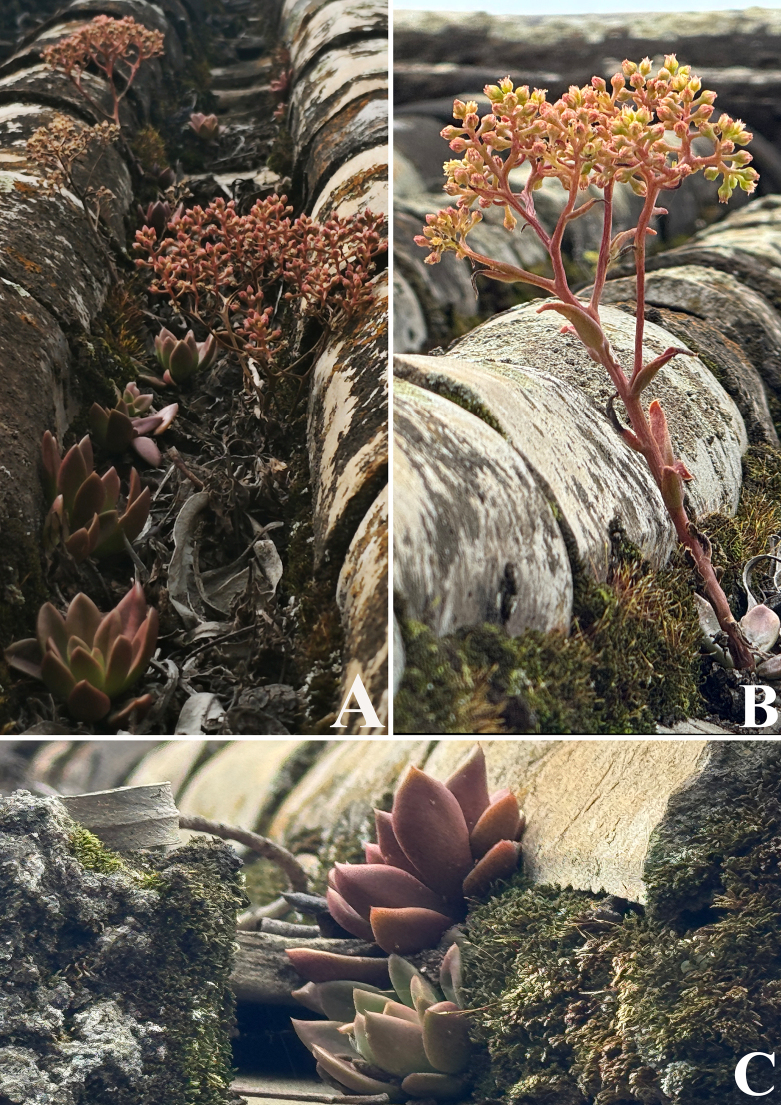
*Sinocrassula chenggongensis*. **A, C**. Habitat; **B**. Habit.

Many species of *Sinocrassula* are mainly distributed in the Himalaya-Hengduan Mountains and adjacent regions (Fu and Ohba 2013; Zhao et al. 2025, 2026), where broad-scale environmental factors, including monsoons, climatic heterogeneity, and river systems, may contribute to geographic and ecological isolation among populations and species (Martíne et al. 2022; Sun et al. 2022). However, these factors alone may not fully explain species diversification and the maintenance of species boundaries in *Sinocrassula*. The two new species described here occur within a radius of 200 m around Santai Mountain, but they are clearly distinguished from each other by several morphological characters (Figs 2, 3, 4, 5). Their close geographic proximity suggests that geographic isolation alone cannot explain their differentiation and persistence as distinct species. Local-scale processes, such as microhabitat differentiation, ecological specialization, and reproductive isolation, may therefore be involved. Previous reports have shown that some species of *Sinocrassula* occur in highly localized habitats, including artificial habitats such as rooftops (Giagnacovo et al. 2025). Such habitats may influence local population structure, dispersal, and contact among closely related species (Hendrickson and Cruzan 2024). In addition, previous studies have reported phylogenetic discordance among datasets in *Sinocrassula* and suggested that hybridization and incomplete lineage sorting may have contributed to this discordance (Zhao et al. 2025, 2026). Future studies based on population-level genomic data, ecological observations, and reproductive biology are needed to assess local ecological differentiation, reproductive isolation, and gene flow, including hybridization, and to clarify how species boundaries are maintained in this genus.

### Taxonomic treatments

#### Sinocrassula kunmingensis

Taxon classificationPlantaeSaxifragalesCrassulaceae

J.Guan Wang & Jing Zhao sp. nov.

F653B115-AC08-5A7E-93A1-8F86475629BC

urn:lsid:ipni.org:names:77393351-1

Figs 2, 3

##### Type.

China • Yunnan: Kunming City, Chenggong District, Santai Mountain; elev. ca. 1933 m; 24.894500, 102.795700; 15 October 2025; *Jia-Guan Wang, Jing Zhao & Chuan-Jie Huang YUS15601* (holotype YUKU!; isotype YUKU!).

##### Diagnosis.

*Sinocrassula kunmingensis* is morphologically similar to *S. obliquifolia* Jing Zhao, J.Guan Wang & X.M. Zhou and *S. crassifolia* Chao Chen, Jing Zhao & J.Guan Wang, but can be distinguished by the following characters. Compared to *S. obliquifolia*, this species stem leaves are greenish-yellow or red (vs. purple-red in *S. obliquifolia*), bracts are ovate-lanceolate (vs. lanceolate in *S. obliquifolia*), nectar scales are subligulate, measuring 0.5–0.8 × 0.4–0.8 mm (vs. rectangular, 0.3–0.5 × 0.2–0.3 mm in *S. obliquifolia*). Compared to *S. crassifolia*, inflorescences are longer, 10–15 cm (vs. 3–10 cm in *S. crassifolia*), flowers are reddish-purple (vs. pinkish-white in *S. crassifolia*), nectar scales are subligulate (vs. reniform in *S. crassifolia*).

##### Description.

Terrestrial or lithophytic, perennial, 25.0–33.0 cm tall, glabrous, rosette 4.0–6.0 × 3.0–6.0 cm in diam. ***Roots*** fibrous. ***Basal leaves*** rosulate, ca. 25–35, spirally arranged, base green or glaucous green, asymmetrically long-spatulate 1.5–3.5 × 0.7–1.0 cm, upward red brown with spots. ***Flowering stems*** terminal, 10.0–15.0 cm, slender, glabrous, red brown with spots, basally branched. ***Stem leaves*** alternate, greenish-yellow or red without spots, lanceolate, relatively narrow and elongated, 2.8–3.2 × 0.7–1.0 cm, obliquely ascending or slightly reflexed. Inflorescence corymbiform, ca. 2.0–3.0 cm in diam, many flowered. ***Bracts*** spreading, ovate-lanceolate, 0.7–1.2 × 0.2–0.4 cm. ***Flowers*** small, ca. 4.0–6.0 mm in diam, pedicels green, slightly longer than flowers. ***Sepals*** broadly triangular, green with purplish-red spots, 1.5–3.0 × 0.5–1.2 mm, minutely papillate, apex acute to acuminate, base truncate to slightly rounded. ***Petals*** triangular, base yellow white, middle with a slight central protrusion, densely purplish-red spotted or streaked upward, 2.0–3.0 × 1.0–2.0 mm. ***Stamens*** slightly shorter than petals, ca. 1.8–2.5 mm long, filaments basally gradually broadened; anthers golden yellow. Nectar scales nearly ligulate, ca. 0.5–0.8 × 0.4–0.8 mm. ***Carpels*** five, lanceolate, arranged clockwise, 2.0–3.0 × 0.5–1.0 mm. Styles slender, white, ca. 1.0–1.5 mm. ***Pollen grain*** prolate-spheroidal, 20.0–25.0 × 10.0–15.0 μm, surface ornamentation irregularly striate. Flowering from September to January.

##### Distribution and habitat.

*Sinocrassula kunmingensis* is currently known in central Yunnan Province, China. Two populations were found in granite crevices, as well as on dry stony or gravelly slopes at elevations ranging from 1933 to 2186 m.

##### Additional specimens examined (paratypes).

China • Yunnan: Kunming City, Wuhua District, Qiongzhu Temple, Yu’an Mountain, elev. ca. 2186 m, 25.0814°N, 102.6272°E, 15 June 2023, *Jing Zhao & Mei-Ying Wu YUS15397* (YUKU!).

##### Etymology.

The epithet *kunmingensis* refers to Kunming City, the location where the species was discovered. Its Chinese name is suggested as ‘昆明石莲 (kun ming shi lian)’.

#### Sinocrassula chenggongensis

Taxon classificationPlantaeSaxifragalesCrassulaceae

J.Guan Wang & Jing Zhao sp. nov.

B4856741-6C5F-573C-B55E-5C258689D849

urn:lsid:ipni.org:names:77393352-1

Figs 4, 5

##### Type.

China • Yunnan: Kunming City, Chenggong District, Santai Mountain Park; elev. ca. 1905 m; 24.898200, 102.793500; 15 October 2025; *Jia-Guan Wang, Jing Zhao & Chuan-Jie Huang YUS15602* (holotype YUKU!; isotype YUKU!).

##### Diagnosis.

*Sinocrassula chenggongensis* is morphologically similar to *S. adpressa*, *S. yongshengensis* Chao Chen & J.Guan Wang and *S. kunmingensis*, but can be distinguished by the following character combinations. Compared to *S. adpressa*, this species has oblong bracts with an oblique apex (vs. elliptic in *S. adpressa*), nectar scales are subligulate, measuring 0.5–0.8 × 0.4–0.6 mm (vs. rectangular, 0.5–0.7 × 0.3–0.5 mm in *S. adpressa*), petals without a protrusion (vs. with a protrusion at the middle in *S. adpressa*), and flowers are pinkish-purple (vs. reddish-purple in *S. adpressa*). Compared to *S. yongshengensis*, this species bracts are oblong bracts with an oblique apex (vs. spatulate or ovate-oblong in *S. yongshengensis*), flowers are pinkish-purple (vs. white to green in *S. yongshengensis*), nectar scales are subligulate (vs. rectangular in *S. yongshengensis*), and petals without a protrusion (vs. with a ridged protrusion at the middle of the upper part in *S. yongshengensis*). Compared to *S. kunmingensis*, this species bracts are oblong bracts with an oblique apex (vs. ovate-lanceolate in *S. kunmingensis*), flowers are pinkish-purple (vs. reddish-purple in *S. kunmingensis*), stem-leaves are green with purplish-red spots (vs. greenish yellow or red without spots in *S. kunmingensis*), and petals without a protrusion (vs. central with a slight protrusion in *S. kunmingensis*).

##### Description.

Terrestrial or lithophytic, perennial, 16.0–24.0 cm tall, glabrous, rosette 5.0–8.0 cm in diam. ***Roots*** fibrous. ***Basal leaves*** rosulate, ca. 20–30, spirally arranged, base green, asymmetrically ovate to lanceolate, 2.5–5.0 × 1.0–2.5 cm, upward purple red with spots. ***Flowering stems*** terminal, slender, ca. 12.0–15.0 cm, glabrous, purple red with spots, unbranched or branched near base. ***Stem leaves*** alternate, green with purplish-red spots, lanceolate, 3.0–4.0 × 1.0–1.5 cm, oblique at base. Inflorescence corymbiform, ca. 2.0–3.0 cm in diam, many flowered. ***Bracts*** oblong-elliptic, apex oblique, densely covered with purple red spots, 0.5–0.7 × 0.2–0.4 cm. ***Flowers*** small, ca. 4.0–6.0 mm in diam, pedicels green, slightly longer than flowers. ***Sepals*** broadly triangular, purple with red spots, 1.5–3.0 × 0.5–1.2 mm, minutely papillate, apex acute to acuminate, base rounded to cuneate. ***Petals*** triangular, without a protrusion, base white, densely pinkish-purple upward, 2.0–3.0 × 1.0–1.5 mm. ***Stamens*** slightly longer than petals, ca. 2.5–3.0 mm, filaments basally gradually broadened; anthers yellow. Nectar scales nearly ligulate, ca. 0.5–0.8 × 0.4–0.6 mm. ***Carpels*** five, lanceolate, arranged clockwise, 2.5–3.0 mm. Styles slender, white, ca. 1.0–1.2 mm. ***Pollen grain*** prolate-spheroidal, 20.0–25.0 × 10.0–15.0 μm, surface ornamentation striate-rugulate. Flowering from September to January.

##### Distribution and habitat.

*Sinocrassula chenggongensis* is currently known in central Yunnan Province, China. One population was found growing on tiles and walls of old houses in villages, as well as on shaded rock faces and boulders in the wild, at an elevation of ca. 1900 m.

##### Etymology.

The epithet *chenggongensis* refers to Chenggong District, the location where the species was discovered. Its Chinese name is suggested as ‘呈贡石莲 (cheng gong shi lian)’.

## Supplementary Material

XML Treatment for Sinocrassula kunmingensis

XML Treatment for Sinocrassula chenggongensis

## Additional information

### Artificial Intelligence (AI) use

The authors accept full responsibility for the content of the manuscript, including the disclosure of any use of AI.

No AI tools were used in the preparation of this manuscript.

### Author ORCIDs

S.-R. Gong https://orcid.org/0009-0006-7231-1352

J. Zhao https://orcid.org/0000-0001-7871-2209

J.-R. Zhang https://orcid.org/0009-0009-0356-4462

S.-F. Liang https://orcid.org/0009-0000-1132-5933

L.-N. Wei https://orcid.org/0009-0002-7101-1973

X.-M. Zhou https://orcid.org/0000-0003-3555-7784

J.-G. Wang https://orcid.org/0000-0003-0264-9180

### Data availability

All of the data that support the findings of this study are available in the main text.
